# The novel p.Cys65Tyr mutation in *NR5A1* gene in three 46,XY siblings with normal testosterone levels and their mother with primary ovarian insufficiency

**DOI:** 10.1186/1471-2350-15-7

**Published:** 2014-01-10

**Authors:** Helena Campos Fabbri, Juliana Gabriel Ribeiro de Andrade, Fernanda Caroline Soardi, Flávia Leme de Calais, Reginaldo José Petroli, Andréa Trevas Maciel-Guerra, Gil Guerra-Júnior, Maricilda Palandi de Mello

**Affiliations:** 1 Centro de Biologia Molecular e Engenharia Genética (CBMEG), Universidade Estadual de Campinas (UNICAMP), Avenida Cândido Rondon 400, 13083-875, Campinas, SP Brasil; 2 Departamento de Genética Médica, Faculdade de Ciências Médicas (FCM) – Universidade Estadual de Campinas (UNICAMP), Rua Tessália Vieira de Camargo 126, 13083-887, Campinas, SP, Brasil; 3 Departamento de Pediatria/Centro de Investigação em Pediatria (CIPED), Faculdade de Ciências Médicas (FCM) – Universidade Estadual de Campinas (UNICAMP), Rua Tessália Vieira de Camargo 126, 13083-887, Campinas, SP, Brasil

**Keywords:** Disorders of sex development, *NR5A1* mutation, Primary ovarian insufficiency

## Abstract

**Background:**

Disorders of sex development (DSD) is the term used for congenital conditions in which development of chromosomal, gonadal, or phenotypic sex is atypical. Nuclear receptor subfamily 5, group A, member 1 gene (*NR5A1*) encodes steroidogenic factor 1 (SF1), a transcription factor that is involved in gonadal development and regulates adrenal steroidogenesis. Mutations in the *NR5A1* gene may lead to different 46,XX or 46,XY DSD phenotypes with or without adrenal failure. We report a Brazilian family with a novel *NR5A1* mutation causing ambiguous genitalia in 46,XY affected individuals without Müllerian derivatives and apparently normal Leydig function after birth and at puberty, respectively. Their mother, who is also heterozygous for the mutation, presents evidence of primary ovarian insufficiency.

**Case presentation:**

Three siblings with 46,XY DSD, ambiguous genitalia and normal testosterone production were included in the study. Molecular analyses for *AR*, *SRD5A2* genes did not reveal any mutation. However, *NR5A2* sequence analysis indicated that all three siblings were heterozygous for the p.Cys65Tyr mutation which was inherited from their mother. *In silico* analysis was carried out to elucidate the role of the amino acid change on the protein function. After the mutation was identified, all sibs and the mother had been reevaluated. Basal hormone concentrations were normal except that ACTH levels were slightly elevated. After 1 mcg ACTH stimulation test, only the older sib showed subnormal cortisol response.

**Conclusion:**

The p.Cys65Tyr mutation located within the second zinc finger of DNA binding domain was considered deleterious upon analysis with predictive algorithms. The identification of heterozygous individuals with this novel mutation may bring additional knowledge on structural modifications that may influence *NR5A1* DNA-binding ability, and may also contribute to genotype-phenotype correlations in DSD. The slightly elevated ACTH basal levels in all three patients with 46,XY DSD and the subnormal cortisol response after 1 mcg ACTH stimulation in the older sib indicate that a long-term follow-up for adrenal function is important for these patients. Our data reinforce that *NR5A1* analysis must also be performed in 46,XY DSD patients with normal testosterone levels without *AR* mutations.

## Background

Steroidogenic factor 1 (SF1), denominated as nuclear receptor subfamily 5 group A member 1 (NR5A1 [OMIM + 184757]), is a protein that regulates several steps of adrenal and gonadal development [1,2]. It is encoded by the *NR5A1* gene, which is an autosomal gene mapped to 30 kb within 9q33. The *NR5A1* gene has one non-translated exon (exon 1), six coding exons (exon 2–7) and six introns [3,4]. The SF1 protein has 461 amino acids divided in two zinc-finger DNA-binding domains (DBD), a ligand-binding domain (LBD), two functional activation domains (AF-1 and AF-2), an accessory region, and a hinge region [5]. SF1 is extremely conserved among species and it presents 95% overall amino acid identity between human and mouse sequences [6].

*NR5A1* is expressed in the developing urogenital ridge, steroidogenic tissues (such as gonads, adrenals, and placenta), hypothalamus and anterior pituitary [7-9]. In general, it activates the expression of AMH in Sertoli cells leading to the regression of Müllerian structures [1,2,9]; in Leydig cells, it activates the expression of several enzymes involved in steroidogenesis, resulting in the virilization of external genitalia and testicular descent [1,2,9]; and, in ovaries, *NR5A1* is expressed in the granulosa and theca cells where it regulates genes required for ovarian steroidogenesis and follicle growth maturation [8,9]. Genes such as: *CYPs*, *HSD3B2, StAR, SOX9, NR0B1*, and others are among the gene targets subject to *SF1* transcriptional regulation [5]. As an essential transcription regulator for adrenal and gonadal development, *SF1* is very important in sex differentiation processes, although it also plays important physiological roles in the central nervous system [10]. Therefore, mutations in *NR5A1* may lead to Disorders of Sex Development (DSD) defined as incomplete or disordered gonadal or genital development, causing divergences between genetic sex, gonadal sex and phenotypic sex [11,12].

p.Gly35Glu and p.Arg92Gln were the first two mutations described in human *NR5A1*. They had been identified in patients with primary adrenal insufficiency, complete gonadal dysgenesis and Müllerian duct persistence [13,14]. Over 50 mutations have been reported mainly in 46,XY DSD individuals with apparently normal adrenal function, but they were also found in 46,XX individuals with primary ovarian insufficiency and normal female phenotype and male individuals with infertility [15-19]. In addition, several reports demonstrated that *NR5A1* variations might be associated with hypospadias, anorchia, and with some cases of adrenal tumors and endometriosis [20,21].

Such findings indicate a complex phenotype expressivity, with different penetrance and variable inheritance pattern for *NR5A1* mutations. Therefore, it is difficult to establish a direct phenotype-genotype correlation [22]. Recently, some authors have reported heterozygous loss-of-function *NR5A1* mutations in patients with clinical features of androgen insensitivity syndrome (AIS) and apparently normal Leydig and Sertoli cell function but without mutations in the androgen receptor gene (*AR*) [23-25].

In this report, we describe the novel c.195G > A *NR5A1* mutation identified in three siblings with 46,XY DSD. They were born of non-consanguineous parents, and had been brought to medical care due to ambiguous genitalia. All of them had normal testosterone levels in first months of life and the proband had normal male puberty. Molecular analyses showed that the putative p.Cys65Tyr missense was inherited from the mother, who presented with signs of primary ovarian insufficiency.

## Case presentation

### Case report

The study was undertaken under an institutionally approved ethic protocol and informed consent was obtained from all subjects and relatives.

**Figure 1:**
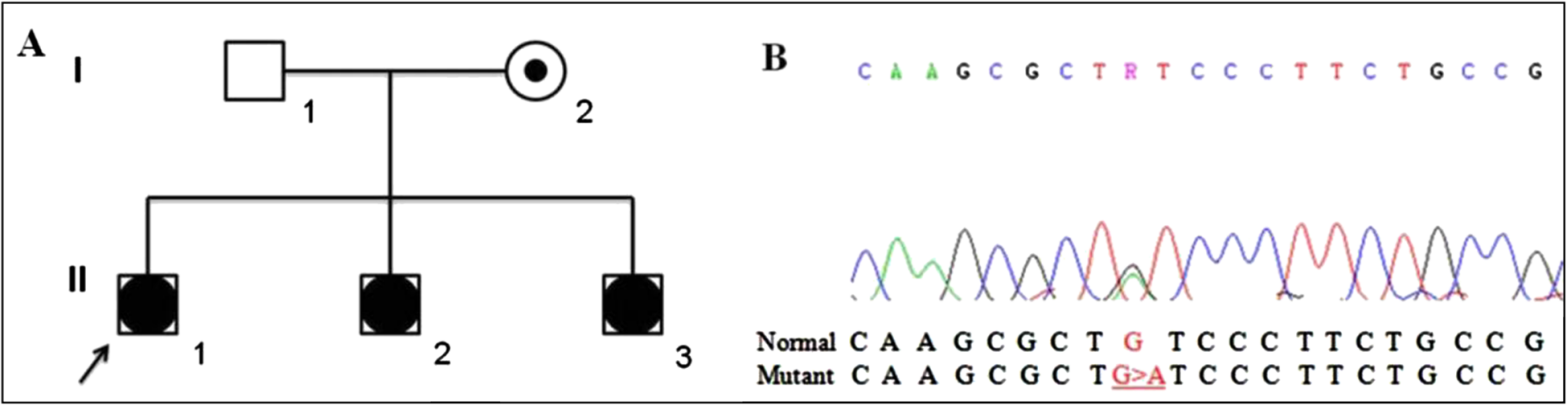
**Pedigree of the family and part of *NR5A1 *exon 3 sequencing. A)** Three siblings and the mother carry the mutation c.195G > A. **B)** Electropherogram showing the c.195G > A heterozygous transition leading to p.Cys65Tyr mutation.

Three affected siblings with 46,XY DSD had been evaluated (Figure 1A). The index case, now aged 15, was born at term to healthy non-consanguineous parents, after an uneventful pregnancy. He was assigned and registered as female at birth and was referred to our service due to ambiguous genitalia at the age of 9 months. Physical examination revealed a 2-cm phallus, a single perineal opening, and palpable gonads in the labioscrotal folds. Laboratory data indicated high levels of FSH but normal levels of LH, and a normal testosterone response to hCG test. The karyotype was 46,XY and pelvic ultrasound showed absence of mullerian derivatives. The medical team presented such results to parents, who also received psychological support. A few months later they decided for female to male sex reassignment. The hypospadias repair was performed when he was 1 year and 4 months old. Puberty began spontaneously when he was 11 years old. Hormone measurements have been performed every year and the concentrations were always normal and high for testosterone and FSH, respectively; whereas, LH levels, that were initially normal, have progressively increased (Table 1). Currently, he is at Tanner stage 4 without hormone replacement therapy. His height is near the target. Recent evaluation of the adrenal function indicated that basal levels for ACTH and cortisol were, respectively, slightly elevated and normal (Table 1). However, cortisol response was subnormal after stimulation with 1 mcg ACTH (Table 1).

**Table 1:** Hormonal values for the three patients

**Patient, age (yr)**	**Clinical presentation**	**Testosterone (nmol/L)**	**FSH (IU/L)**	**LH (IU/L)**	**Estradiol (nmol/L)**	**Cortisol (nmol/L)**	**ACTH (pmol/L)**
**Patient 1**							
**0.75**	Micropenis and perineal hypospadia	1.04 (pre hCG) 6.59 (after hCG)	11.90	2.80	-	-	-
**11**	Started spontaneuous puberty				-		
**13**	Tanner IV	12.87	27.25	11.96	-		
**14**	Tanner IV-V	15.89	23.51	10.95	-	376.46	7.08
**15**	Tanner IV-V	16.79	21.60	16.13	-	261.80	16.10
						421.60*	
**Patient 2**							
**0.17**	Micropenis and penoscrotal hypospadia	7.63	4.85	10.00	-	-	-
**6**	Tanner I	0.66	1.15	<0.10	-	576.01	7.06
**7**	Tanner I	0.66	1.51	<0.10	-	195.61	14.87
						572.49*	
**Patient 3**							
**0.17**	Micropenis, perineal urogenital opening and bilateral cryptorchidism	0.66 (pre hCG) 6.94 (after hCG)	4.62	0.20	-	-	-
**5**	Tanner I	-	-	-	-	820.27	9.99
**6**	Tanner I	0.66	2.60	<0.10	-	300.21	12.61
						613.10*	
**Mother**							
**32**	Irregular menses	-	13.81	7.05	176.22	218.31	7.37
**33**	Irregular menses	-	48.88	19.83	109.11	388.70	9.66
						645.33*	
**Normal range**		Newborn: 2.43-13.88	Male: 1.50-12.40 Female (menopause): 25.80-134.80 Children: 0.20-3.80	Male: 1.70-8.60 Female (menopause): 7.70-58.85 Children: 0.20-1.40	Female (menopause): <200.75	8 a.m.: 138.00-690.00 ≥ 551.80*	<10.12
		Children: <5.89					
		Puberty:					
		Tanner I - <0.69					
		Tanner II - <14.92					
		Tanner III – 2.25- 27.07					
		Tanner IV – 6.47-26.37					
		Tanner V – 6.59-30.54					
		Male 18–49 yrs: 8.64-29.01					

*after 1 mcg ACTH test.

The second sib, currently aged 7 years, was born at term after an uneventful pregnancy. At birth he presented 2-cm phallus, penoscrotal hypospadias and palpable gonads in the labioscrotal folds. His karyotype was 46,XY. Pelvic ultrasound did not show mullerian derivatives. Hormone investigation was performed at 2 month of age and resulted in high LH level with normal levels of FSH and testosterone (Table 1). He was assigned as male and had the hypospadias repaired. In a recent evaluation of the adrenal function, basal levels of ACTH and cortisol were, respectively, slightly elevated and normal (Table 1). Upon 1 mcg ACTH stimulation, cortisol response was normal (Table 1).

The third sib, who is currently 6 years old, was born at term after an uneventful pregnancy with a 1.3-cm phallus, a single perineal urogenital opening, and palpable gonads, both in the inguinal region. He also had a normal 46,XY karyotype. Pelvic ultrasound did not show mullerian derivatives. At the age of 2 months, hormone levels for FSH, LH, testosterone and dihydrotestosterone were normal (Table 1). He was assigned as male and underwent hypospadias repair and orchidopexy. Recent evaluation of the adrenal function revealed slightly elevated basal ACTH and normal basal cortisol levels (Table 1). He also had a normal cortisol response upon 1 mcg ACTH stimulation test (Table 1).

The 33-year-old mother had throughout hormone evaluation after identifying the *NR5A1* mutation (see below). Hormonal results revealed high FSH, normal to high LH and normal to low estradiol levels with normal adrenal function (Table 1). She also reported irregular menses and hot flushes, suggesting primary ovarian insufficiency.

## Methods

Genomic DNAs from patients and parents were purified from peripheral leukocytes by proteinase K lysis, phenol/chloroform extraction, and ethanol precipitation, according to standard techniques. Sequencing of both *AR* (androgen receptor) and *SRD5A2* (5α-reductase) genes had been performed as described elsewhere [26,27]. The *NR5A1* exons and 5’ and 3’ untranslated flanking regions were amplified by polymerase chain reaction (PCR) using specific primers designed based on the normal gene sequence (ENSG00000136931, http://www.ensembl.org). Independent PCR fragments were purified in 1% agarose gel electrophoresis with the Wizard SV Gel and PCR clean-up system (Promega, Madison, WI, USA), and both sense and antisense strands were sequenced using the BigDye Terminator v3.1 Cycle Sequencing Kit (Life Technologies, Grand Island, NY, USA) with the same primers used in PCR reactions. The Chromas Lite 2.0 (Technelysium Pty Ltd) and CLC Sequence Viewer v.6.8.1 free software (CLC bio) were used to analyze and compare sequences with the reference *NR5A1* sequence. Structural analyses were performed using PDB ID: 2FF0 – chain A as template. The native and mutant models were constructed by SWISS MODEL web-served program. Internal contacts were evaluated by STING Millenium (http://www.cbi.cnptia.embrapa.br) and visualized by PyMol®.

## Results

DNA sequence analyses of *AR* and *SRD5A2* genes did not show any mutation. However, *NR5A1* gene sequencing revealed a novel heterozygous transition G > A within exon 3 in all three siblings as well as in their mother (Figure 1B). The nucleotide change c.195G > A is predicted to cause the substitution of a cysteine by a tyrosine at the amino acid residue 65 (p.Cys65Tyr).

**Figure 2:**
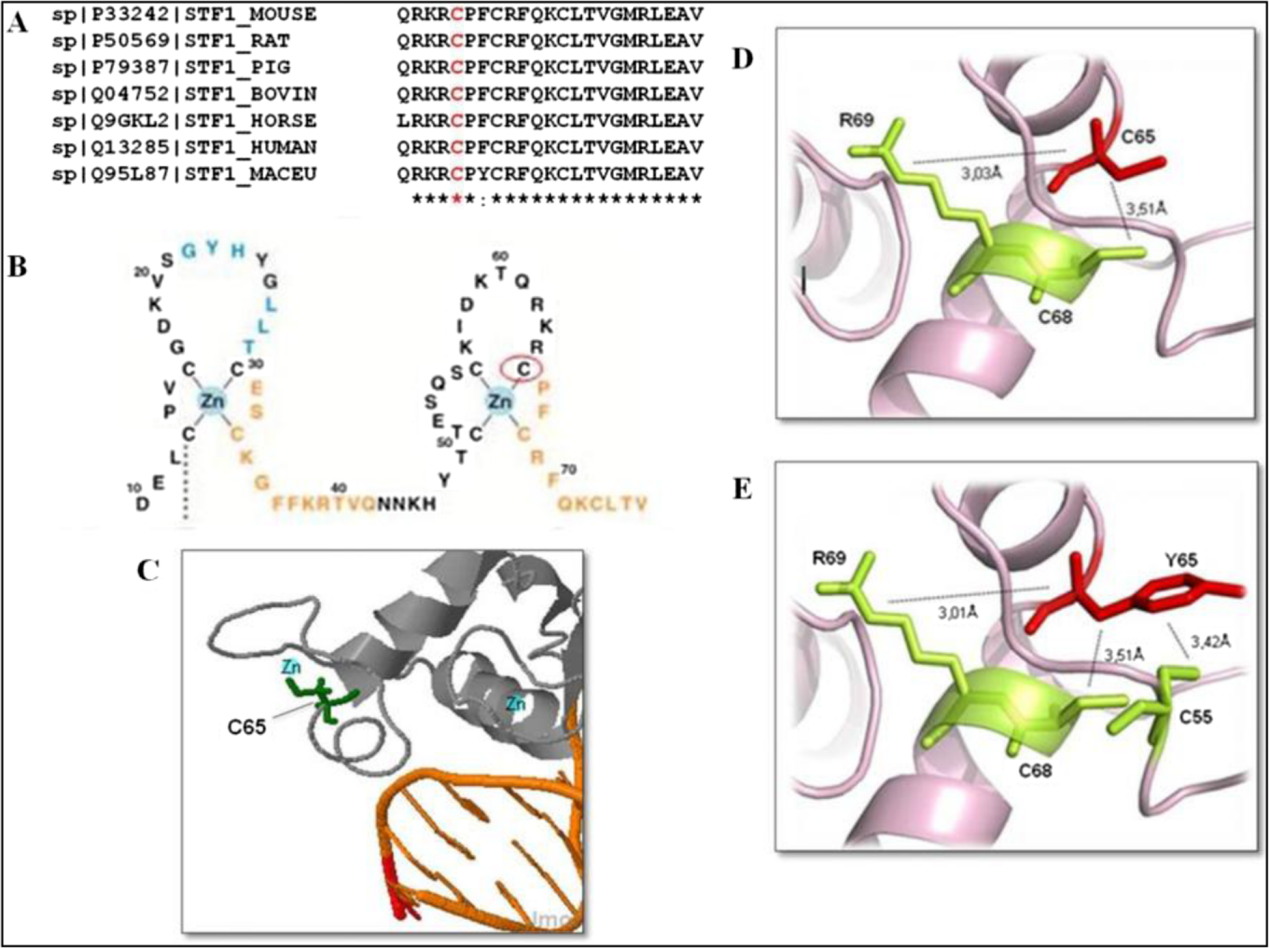
**Comparison of normal and mutant NR5A1 protein considering sequence conservation and internal aminoacid interactions. A)** Multiple alignment of NR5A1 protein family using ClustalW: the conserved residue C65 is shown in red. **B)** Scheme of the two zinc fingers from the DNA-binding domain (DBD). Red circle denotes the C65 residue (adapted from Little *et al*. [25]). **C)** Structural complex of NR5A1 bound to DNA showing the C65 residue ligated to the zinc atom at the zinc-finger binding site within the DNA binding domain. **D)** Structural model of the native protein showing internal contacts. The C65 interacts by hydrogen bond with R69 and hydrophobic interaction with C68. **E)** Mutant protein internal contacts. The Y65 establish new hydrophobic contact with C55.

The cysteine residue in position 65 in the NR5A1 protein is highly conserved across mammalian species (Figure 2A). It is located at the second zinc finger of DNA binding domain, as illustrated by Little *et al*. [28] (Figure 2B, C). Structural analyses demonstrated that C65 in the native protein makes a hydrogen bond with R69 and has a hydrophobic interaction with C68 (Figure 2D). The mutant Y65 maintains both interactions. However, a new hydrophobic interaction by 3.42 Ångstroms is established with C55 (Figure 2E).

Three predictive methods to evaluate the effect of the amino acid substitution were used: PolyPhen (Polymorphism Phenotyping) that gives scores ranging from 0 (neutral) to a positive (damaging) number; SIFT (Sorting Intolerant From Tolerant) whose scores range from 0 (damaging) to 1 (neutral); and Aling GV-GD that classifies the amino acid change into classes ranging from C0 to C65, where C0 is considered tolerant and C65 deleterious [29,30]. The p.Cys65Tyr mutation resulted in PolyPhen score of 1.0, SIFT score of 0 and Aling GV-GD put it into class C65 indicating a protein damage, probably leading to patients’ phenotypes. In order to discard the possibility of the nucleotide variation being a frequent polymorphism, 86 healthy controls (172 alleles) were analyzed and c.195G > A was not identified in any allele.

## Discussion

We present here the follow-up of patients with 46,XY DSD in a Brazilian family. The three sibs presented with different hormone profiles in the first year of life. Testosterone levels were normal for all of them; however, sib 1 and sib 2 presented isolated elevation of FSH and LH levels, respectively, whereas sib 3 had normal levels for both FSH and LH. Initially, *AR* and *SRD5A2* gene sequence analyses had been performed due to 46,XY karyotype, ambiguous genitalia without Müllerian derivatives and normal production of testosterone. As mutations in those genes had not been identified, patients remained idiopathic and the etiologic diagnosis was not defined, even though the severity of ambiguous genitalia and the familial recurrence clearly indicated a genetic origin. After long-term follow-up, the etiology of 46,XY DSD had been solved by the identification of p.Cys65Tyr mutation in the *NR5A1* gene which was investigated based on the recent description of variable phenotypic expression as a result of different *NR5A1* mutations, and also based on the possibility of a mutation inheritance from a fertile mother, which mimicked an X-linked recessive pattern [23-25].

Most 46,XY patients with *NR5A1* loss-of-function mutations have had biological markers that evidenced gonadal dysgenesis, i.e.: LH and FSH levels were usually elevated, AMH levels, if measured, were low, and Leydig cell function, indicated by low levels of testosterone, was invariably defective [23]. Testosterone levels in the three patients were normal during the first year of life and puberty development of the proband also indicated normal Leydig cell function after birth. In addition, Müllerian derivatives were not found suggesting that Sertoli cell function was also normal during primary sex differentiation. As discussed by Tantawy *et al.*[31], there are very few reports on 46,XY DSD cases that had puberty development and normal male testosterone production inducing spontaneous virilization, however long-term follow-up indicated a progressive gonadal failure with elevated FSH in such cases. Although the proband described here developed normal puberty, he persisted with high levels of FSH from first months of life till puberty suggesting some degree of tubular defect that might cause fertility impairment.

Recently, Wu *et al*. [25] speculated if SF1 disruption caused by *NR5A1* loss-of-function mutations may be associated with functional androgen resistance or altered Leydig cell maturation leading to hyper-responsiveness to postnatal LH stimulation. Our data also suggest such a mechanism and reinforce that, in the absence of *AR* mutations, *NR5A1* gene analysis must be performed in 46,XY DSD despite normal testosterone levels.

Several mutations have been described in *NR5A1* in different cases of 46,XY DSD so far [15,17,32,33], however a mutation in the C65 residue has been described here for the first time. The nucleotide change c.195G > A results in p.Cys65Tyr missense which is predicted as damaging by *in silico* tools. It is well known that cysteine influences the overall three-dimensional structure of proteins. Its sulfur group reacts quite readily with other sulfur groups, forming disulfide bonds that play important role in the folding and stability of proteins. Cysteine residues also play a valuable role in crosslinking proteins, which increases protein rigidity and also confers proteolytic resistance [34]. Conversely, tyrosine is an aromatic and partially hydrophobic amino acid [34]. The aromatic side chain is usually involved in stacking interactions with other aromatic side chains [34]. Tyrosine can also be involved in phosphorylation within intracellular proteins [34]. In the structural analyses, a previously inexistent hydrophobic interaction with C55 was observed for Y65 residue. In addition, this novel mutation is located within the second zinc finger of DNA binding domain where C65 itself binds directly to the zinc atom [28], therefore the change to tyrosine may influence *NR5A1* DNA-binding ability by destabilizing zinc-finger conformation. Although functional analyses will be further necessary for formal demonstration of a deleterious effect for p.Cys65Tyr mutation, both predictive and structural analyses indicate that it might correlate with the DSD phenotype in the three heterozygous siblings. Their fertile 33-year-old mother who is also heterozygous for the mutation reported irregular menses and hot flushes, after molecular diagnosis. Upon endocrine investigation, gonadotropin and estradiol levels suggest primary ovarian insufficiency. Taken together, those data indicate that p.Cys65Tyr mutation may also compromise the ovarian functional maintenance similar to other *NR5A1* mutations described in the literature [15-17].

Considering the slightly elevated basal ACTH levels in all three patients with 46,XY DSD and the subnormal cortisol response after stimulation test with 1 mcg ACTH in the proband, it may be inferred that p.Cys65Tyr mutation could have a late-onset effect upon adrenal function, justifying a long term follow-up on such patients.

## Conclusion

In conclusion, based on recent knowledge concerning the phenotypic expression of *NR5A1* mutations, the analysis of this gene becomes an important tool not only for diagnosing patients with DSD including the cases with normal testosterone secretion without *AR* mutations, but also for identifying their female relatives at risk of developing primary ovarian insufficiency and allowing reproductive counseling as well as potentially assisted reproductive techniques.

## Consent

Written informed consent was obtained from each member of the family for publication of this Case Report and any accompanying images. A copy of the written consent is available for review by the Series Editor of this journal.

## Competing interests

The authors declare that they have no competing interests.

## Authors’ contributions

HCF carried out the molecular genetic studies with *NR5A1* gene, participated in the *NR5A1* sequence alignment and drafted the manuscript. JGRA carried out the clinical genetic studies and contributed with writing clinical description of the cases for the manuscript draft. FCS contributed with the structural analysis of normal and mutant proteins. FLC conducted the molecular genetic studies with *SRD5A2* gene and sequence alignment investigation. RJP was responsible for the molecular genetic studies with *AR* gene and sequence alignment comparison. ATM-G participated in the design of the study and was responsible for the clinical genetic evaluation of the patients. GG-J participated in the design of the study and was responsible for the endocrine evaluation of the patients. MP-de-M conceived of the study, and participated in its design and coordination and helped to draft the manuscript. All authors read and approved the final manuscript.

## Authors’ information

Helena Campos Fabbri, Juliana Gabriel Ribeiro de Andrade shared first authorship.

## Pre-publication history

The pre-publication history for this paper can be accessed here:

http://www.biomedcentral.com/1471-2350/15/7/prepub
